# Development, deployment and in-field demonstration of mobile coronavirus SARS-CoV-2 Nucleic acid amplification test

**DOI:** 10.1099/jmm.0.001346

**Published:** 2021-04-15

**Authors:** Teagan F. Paton, Ian Marr, Zoe O’Keefe, Timothy J. J. Inglis

**Affiliations:** ^1^​ Department of Microbiology, PathWest Laboratory Medicine WA, Queen Elizabeth II Medical Centre, Nedlands 6009, WA, Western Australia, Australia; ^2^​ Menzies School of Health, National Health Laboratory, Dili, Timor-Leste; ^3^​ Schools of Medicine and Biomedical Sciences, the University of Western Australia, Crawley 6009 WA, Australia

**Keywords:** SARS-CoV-2, COVID-19, coronavirus, nucleic acid test, RT-PCR, mobile laboratory, molecular diagnostics, diagnostic test performance

## Abstract

**Introduction:**

The evolving SARS-CoV-2 coronavirus pandemic presents a series of challenges to clinical diagnostic services. Many proprietary PCR platforms deployed outside centralised laboratories have limited capacity to upscale when public health demands increase. We set out to develop and validate an open-platform mobile laboratory for remote area COVID-19 diagnosis, with a subsequent field trial.

**Gap Statement:**

In regional Western Australia, molecular diagnostic support is limited to near point-of-care devices. We therefore aimed to demonstrate open-platform capability in a rapidly deployable format within the context of the COVID-19 pandemic.

**Methodology:**

We compared, selected and validated components of a SARS-CoV-2 RT-PCR assay in order to establish a portable molecular diagnostics laboratory. The optimal combination of PCR assay equipment, reagents and consumables required for operation to national standards in regional laboratories was identified. This comprised RNA extraction and purification (QuickGene-480, Kurabo, Japan), a duplex RT-PCR assay (Logix Smart COVID-19, Co-Diagnostics, USA), a Myra liquid handling robot (Biomolecular Systems, Australia) and a magnetic induction thermal cycler (MIC, Biomolecular Systems).

**Results:**

The 95 and 99% limits of detection were 1.01 copies μl^−1^ (5.05 copies per reaction) and 2.80 copies μl^−1^ (14.00 copies per reaction) respectively. The Co-Diagnostics assay amplified both SARS-CoV-1 and −2 RNA but showed no other cross reactivity. Qualitative results aligned with the reference laboratory SARS-CoV-2 assay (sensitivity 100% [95 % CI 96.48–100%], specificity 100% [95% CI 96.52–100%]). In field trials, the laboratory was operational within an hour of arrival on-site, can process up to 36 samples simultaneously, produces results in two and a half hours from specimen reception, and performed well during six consecutive runs during a 1 week deployment.

**Conclusion:**

Our mobile laboratory enables an adaptive response to increased test demand, and unlike many proprietary point-of-care PCR systems, rapid substitution with an alternative assay if gene targets change or reagent supply chains fail. We envisage operation of this RT-PCR assay as a standby capability to meet varying regional test demands under public health emergency operations guidance.

## Introduction

COVID-19 presents a multitude of challenges to clinical laboratory services. This rapidly expanding pandemic highlights the need for agile testing processes which can adapt to unexpected changes in COVID-19 epidemiology. Limits on diagnostic turnaround times hinder important public health responses, and the ability for clinicians to track, test and isolate. The initial mainstay of molecular methods relied upon centralised, reference laboratory services. More recently, proprietary cartridge-based nucleic acid amplification test (NAAT) platforms have been deployed for urgent *in situ* testing in regional centres. These tests, however, create further challenges with higher costs per test and ongoing supply chain difficulties [1]. Furthermore, many of these assays lack the flexible target selection and throughput available with open systems, limiting their utility outside the current pandemic.

We previously showed that regional deployment of an in-house polymerase chain reaction (PCR) assay during the A/H1N1/2009 pandemic expedited diagnosis, treatment and disease control measures [1]. Our ongoing work with successive equipment platforms and optimised reagent chemistry allowed NAAT methods to both enter regional and remote Australia and assist our international neighbours [2–6]. A decreasing equipment payload and more durable molecular reagents have increased the portability of PCR assays, extending the reach of molecular diagnostics closer to the point of care [3, 7]. However, the rapid emergence of novel infectious diseases such as COVID-19 threaten to overwhelm public laboratory services before new assays can be validated for clinical use. Even under the provisions of an emergency use approval, there is substantial additional work to validate a new diagnostic assay [8]. Immediate disruption of the typical laboratory transport chains during the early stages of the current coronavirus pandemic, meant regional Australia was at particular risk of prolonged turnaround times and subsequently poor public health responses. Multiple proprietary cartridge-based NAAT platforms (GeneXpert, Cepheid, Sunnyvale, CA) were deployed to regional Western Australian centres to provide rapid test capability. These had limited capacity to upscale when public health demands increased and highlighted the need for an independent, near-to-care diagnostic solution with greater throughput than existing point-of-care platforms provide.

The first manufacturer-independent evaluation of a potentially portable SARS-CoV-2 reverse transcriptase PCR (RT-PCR) assay system was reported in May [9]. The feasibility of a SARS-CoV-2 assay without an RNA extraction step was considered as a means to reduce costs and improve test portability [10]. However, in order to more closely align with Australian public health laboratory network (PHLN) guidelines and practices [11], we opted to design and validate a mobile molecular diagnostic laboratory for remote area COVID-19 diagnosis that was inclusive of RNA extraction and purification. The system was deployed for field trials into two static PathWest clinical laboratories in north-western Australia. These laboratories, in Derby and Broome, were at a distance of 1807 and 1684 km respectively from the current centralised molecular diagnostic pathology services. Although both sites had adequate biosafety equipment available, neither was equipped for molecular diagnostics, aside from a single GeneXpert IV instrument at the Broome laboratory. This report presents the development, deployment and in-field demonstration of a fully equipped mobile laboratory, configured to perform SARS-CoV-2 RT-PCR assays.

## Methods

In order to establish a robust, compact and portable testing schema, we carried out performance tests on candidate components. This was limited to currently available equipment, reagents and consumables that could be successfully operated to national quality standards (ISO 15189) in regional clinical laboratories, many of which are not equipped for molecular diagnostics.

In summary, this comprised:

1. Extraction methods: QuickGene Tissue II RNA Kit, nucleic acid purification, silica capture with pressure manifold (Kurabo, Japan) versus QuickExtract rapid extraction (Lucigen Corporation, USA).
2. RT-PCR assay: Logix Smart COVID-19 duplex RT-PCR assay (RDRP gene and human RNA control, 400 assays, Co-Diagnostics, USA).
3. Equipment: Class II biosafety cabinet, QuickGene-480 eight-channel pressure manifold; QBD4 dry block heater (Grant Instruments, UK), four channel / 48 sample magnetic induction cycler (MIC) (Bio Molecular Systems, Australia) and Myra liquid handling robot (Bio Molecular Systems, Australia).
4. Samples: SARS-CoV-2 standard quantified on a QX200 Droplet Digital PCR System (Bio-Rad Laboratories); a nucleic acid extract library consisting of 56 commensal and pathogenic viral, bacterial and fungal targets, including endemic coronaviruses and SARS-CoV-1; 207 clinical samples with validated results for SARS-CoV-2 by the PathWest PHLN-aligned in-house developed RT-PCR assay (IHD-RT-PCR) and RdRp sequencing (positive *n*=103, negative *n*=104).

### SARS-CoV-2 culture

We performed viral culture to obtain SARS-CoV-2 RNA for downstream analysis. One hundred microlitres of PCR confirmed SARS-CoV-2 positive swab washings were inoculated into a Nunclon 12-well tissue culture plate (Thermo Fisher Scientific, USA). Each well contained Vero E6 cells in two millilitres of minimal essential media (MEM) modified with 13.3 mM sodium bicarbonate and 25 mM HEPES (PathWest Media, Australia). To reduce the risk of fungal contamination, one millilitre of an 820 mg l^−1^ colloidal suspension of nystatin (Sigma-Aldrich, USA) in Hank’s balanced salt solution (PathWest Media, Australia) was added to every 100 ml MEM for a final concentration of 8.2 mg l^−1^. The inoculated medium was then incubated at 36.5 °C until cytopathic effects were apparent. The viral culture fluid (VCF) was then aspirated, and the SARS-CoV-2 RNA isolated using a QIAamp Viral RNA Mini Kit (Qiagen, Germany).

### SARS-CoV-2 standard RNA quantification

We performed droplet digital PCR (ddPCR) on the SARS-CoV-2 specific E-gene targets in quadruplicate 10^−4^ and 10^−5^ dilutions of the VCF extract to quantify viral copy number. The ddPCR reaction mixes consisted of 1×One Step ddPCR Super Mix (Bio-Rad Laboratories, USA), 20 U µl^−1^ of One-Step reverse transcriptase (RT) (Bio-Rad Laboratories, USA), 15 mM of dithiothreitol (DTT) (Bio-Rad Laboratories, USA), 0.9 µM of forward and reverse primers for the E-gene target, 0.25 µM of probe, and 10 µl of template for a final volume of 30 µl. For droplet generation, the PCR reaction mix was emulsified in 70 µl of droplet generation oil and loaded onto the droplet generator of a QX200 Droplet Digital PCR System using a GCR 96 cartridge (Bio-Rad Laboratories, USA). After droplet generation, each sample was transferred into a new 96-well PCR plate (Axygen, USA) for thermal cycling on a C1000-Touch thermocycler (Bio-Rad Laboratories, USA). Cycling conditions were as follows; 50 °C for 30 min; 95 °C for 10 min; and then 40 cycles of 95 °C for 30 s, 55 °C for 60 s. Cycling was followed by final hold of 98 °C for 10 min. Post-amplification, the plate was loaded onto the droplet reader of the QX200 and the droplets from each well analysed, and the resulting data output analysed with proprietary software (QuantaSoft v1.4.0, Bio-Rad Laboratories, USA).

### Extraction methods

Prior to selection, we compared the relative efficiency between the extraction methods. The sample used for comparison was SARS-CoV-2 VCF, diluted 1 : 100 in viral transfer medium (VTM, PathWest Media, Australia). Both the QuickExtract and QuickGene protocols were carried out as per manufacturer’s instructions [12, 13]. For both extraction methods, three replicates of a two-fold dilution series (undiluted, 50, 25 and 12.5 %; template in molecular grade water) were prepared from replicate SARS-CoV-2 VCF extracts and RT-PCR performed using the Logix Smart COVID-19 assay in order to compare relative Cq values.

### Sensitivity testing

To determine lower limits of detection, we performed PCR on multiple replicates of the diluted SARS-CoV-2 RNA standard. From this original standard, we prepared a 100-fold dilution (30795 copies µl^−1^) in molecular grade water. This working stock was then split into multiple aliquots, and stored at −80 °C until needed. To ensure consistency between runs, aliquots were not re-used. To minimise pipetting errors, all dilutions were prepared using a Myra liquid handling robot. We approached sensitivity testing in two phases. First, we performed a wide range ten-fold serial dilution, from 10^−3^ to 10^−9^, to determine an approximate lower limit of detection. Secondly, we aimed to perform linear dilutions within this range to determine a more precise endpoint.

For the Logix Smart kit, five microlitres of template was added to five microlitres of master mix. The reaction setup was performed with the Myra liquid handling robot running Myra software v.1.6.3. Each dilution of SARS-CoV-2 RNA was replicated ten times. An additional ten replicates were performed of the 10^−6^ dilution (3.1 copies µl^−1^), for a total of twenty replicates, as this concentration was the first to fall beneath the manufacturer’s claimed lower limit of detection. For the second phase, we performed ten replicates each of 20, 10, 8, 6, 4, 1 and 0.5 copies µl^−1^. An additional five replicates each of 0.2 and 0.1 copies µl^−1^ were also performed. Thermal cycling was performed on the MIC with the following cycling conditions: 45 °C for 15 min (reverse transcriptase step), 95 °C for 2 min (initial denaturation), and then 50 cycles of 95 °C for three seconds, 55 °C for 32 s [14]

Data were analysed in MIC software (v.2.8.13, Biomolecular Systems) with the following settings: dynamic analysis, automatic threshold, extensive exclusion. Data were then exported to Microsoft Excel for tabulation. Probit regression (dose response curve – EC_anything_) was performed using statistics software (Prism v8.4.2, GraphPad Software, USA) to determine the lower limits of detection.

### Specificity testing

A panel of 56 samples was selected for cross reactivity testing in the Logix Smart COVID-19 assay. The sample panel consisted of PCR-confirmed nucleic acid extracts from viral, bacterial and fungal organisms. All reactions were set up using the Myra as per sensitivity testing, and cycled on the MIC with manufacturer recommended cycling conditions [14]. For each assay kit, data were acquired across two runs, each containing two replicates of positive and no-template controls. Data were concatenated and analysed in MIC software and exported to Microsoft Excel (Microsoft, USA) for tabulation with the following settings: dynamic analysis, automatic threshold, extensive exclusion.

### In-house developed SARS-CoV-2 RT-PCR assay

We used results obtained from the internally validated PathWest SARS-CoV-2 IHD-RT-PCR as the comparator for this study. The assay comprised duplexed E-gene (envelope protein) and S-gene (spike protein) targets. The E-gene target was pan-Sarbecovirus reactive by design, and subsequently exhibited reactivity with SARS-CoV-1. The S-gene target was specific for SARS-CoV-2, and exhibited no cross reactivity with any other template tested. The 95 % LLoD for SARS-CoV-2 in the proposed S gene duplex real-time RT-PCR assays were previously determined using quantified SARS-COV-2 RNA control material and found to be 10.2 and 17.3 copies per reaction, (E-gene, S-gene respectively), equivalent to 1.28 and 2.16 copies µl^−1^ of RNA extract. All clinical samples assayed by this method were extracted using the MagnaPure 96 instrument (Roche Life Sciences, Penzberg, Germany).

### Co-diagnostics evaluation specimen selection

We selected a panel of 211 historical clinical specimens of known SARS-CoV-2 positivity status. Prior to this work, the samples were suspended in viral transfer media, decanted, tested for SARS-CoV-2 by the IHD-RT-PCR assay, confirmed by sequencing (PathWest Laboratory Medicine, Australia), and stored at −20 °C. As this work was conducted in the early stages of the SARS-CoV-2 pandemic in Australia, we were instructed by PathWest to conserve clinical specimens for use in ongoing test development. In compliance with this requirement, we performed a 1 in 5 dilution on each specimen to be tested by retrieving forty microlitres of sample from each stored aliquot and adding it to 160 µl of sterile viral transfer medium.

We found it necessary to curate the panel in order to compensate for dilution and potential RNA degradation in storage. This facilitated more accurate comparisons between the IHD-RT-PCR and the Logix Smart assays. We consequently implemented the following exclusion algorithm; 1. Samples with a previous IHD-RT-PCR Cq value of equal or greater than 37 in any target were excluded from analysis on the basis that this dilution was likely to reduce viral copy number beneath the claimed limits of detection; 2. Samples arising from clearance testing of PCR-confirmed cases were excluded from analysis due to the differences in internal reporting algorithms, whereby samples returning otherwise equivocal results (Cq=40 or higher) with the IHD-RT-PCR had been reported as positive, potential leading to systematic discrepancies in qualitative results; 3. Any non-dry swab specimens, such as sputa or bronchial washings were to be excluded from this analysis due to additional processing requirements.

A total of four samples met the exclusion criteria. Of the remaining 207 specimens, 152 swabs were obtained from nose and throat swabs, 30 from nasopharyngeal swabs, one from throat only, and 24 from unspecified sites. Of these, 103 specimens were confirmed positive for SARS-CoV-2 by IHD-RT-PCR and DNA sequencing. Three samples were excluded on the basis of late Cq values in the corresponding undiluted specimen used in the IHD-RT-PCR or testing of disease clearance swabs (whereby equivocal result reported as positive). One further sample was excluded on the basis that it was sputum.

### Error discrepancy investigation

The SARS-CoV-2 status of all patient samples was known for this analysis. In the event of discrepant results between the IHD-RT-PCR and the Logix Smart assays, the sample was repeated *de novo* from undiluted specimen in both assays. The data from this repeat testing superseded the data obtained from the initial PCR assay on diluted samples.

### Sample extraction and patient sample validation

All samples were extracted using the QuickGene Tissue RNA II Kit (Kurabo, Japan) processed on the QuickGene-Mini480 manifold with minor modifications to the manufacturer’s protocol [13]. The Kurabo lysis buffer (LRT) was pre-prepared with DTT (Sigma-Aldrich, USA) to a final concentration of 10 mM, 10 ng per ml of carrier RNA (Thermo Fisher Scientific, USA) and approximately 30 copies µl^−1^ of MS2 bacteriophage to serve as a secondary inhibitor control. The DTT was maintained at −20 °C and added to the lysis buffer immediately prior to RNA extraction. To produce the lysate, 150 µl of specimen was added to 200 µl of pre-prepared LRT, mixed thoroughly and allowed to incubate at ambient temperature for 15 min. An extracted negative control was placed after every five patient samples. To this, 185 µl of Kurabo solubilisation buffer (SRT) was added to the lysate and mixed. Finally, 185 µl of 100 % molecular grade ethanol (Rowe Scientific, Australia) was added and mixed. The lysate was then transferred to a corresponding extraction column and pressurised for a minimum of 15 s with the QuickGene-Mini480. Then 600 µl of pre-prepared Kurabo wash buffer (WRT) was added to each column and pressurised for 15 s. This WRT wash was repeated once with the columns pressurised for a minimum of 30 s to ensure all buffer had been removed from the columns. Finally, 100 µl of elution buffer (Kurabo) was dispensed into each extraction column, incubated at ambient temperature for 3 min and pressurised for a minimum of 30 s to elute nucleic acids. PCR reactions were set up using the Myra liquid handler and cycled on the MIC using the recommended cycling conditions with the supplied PC and NTC. Data were acquired across six separate runs. Data were analysed in MIC software and exported to Microsoft Excel for tabulation.

### Field trials

**Fig. 1.**
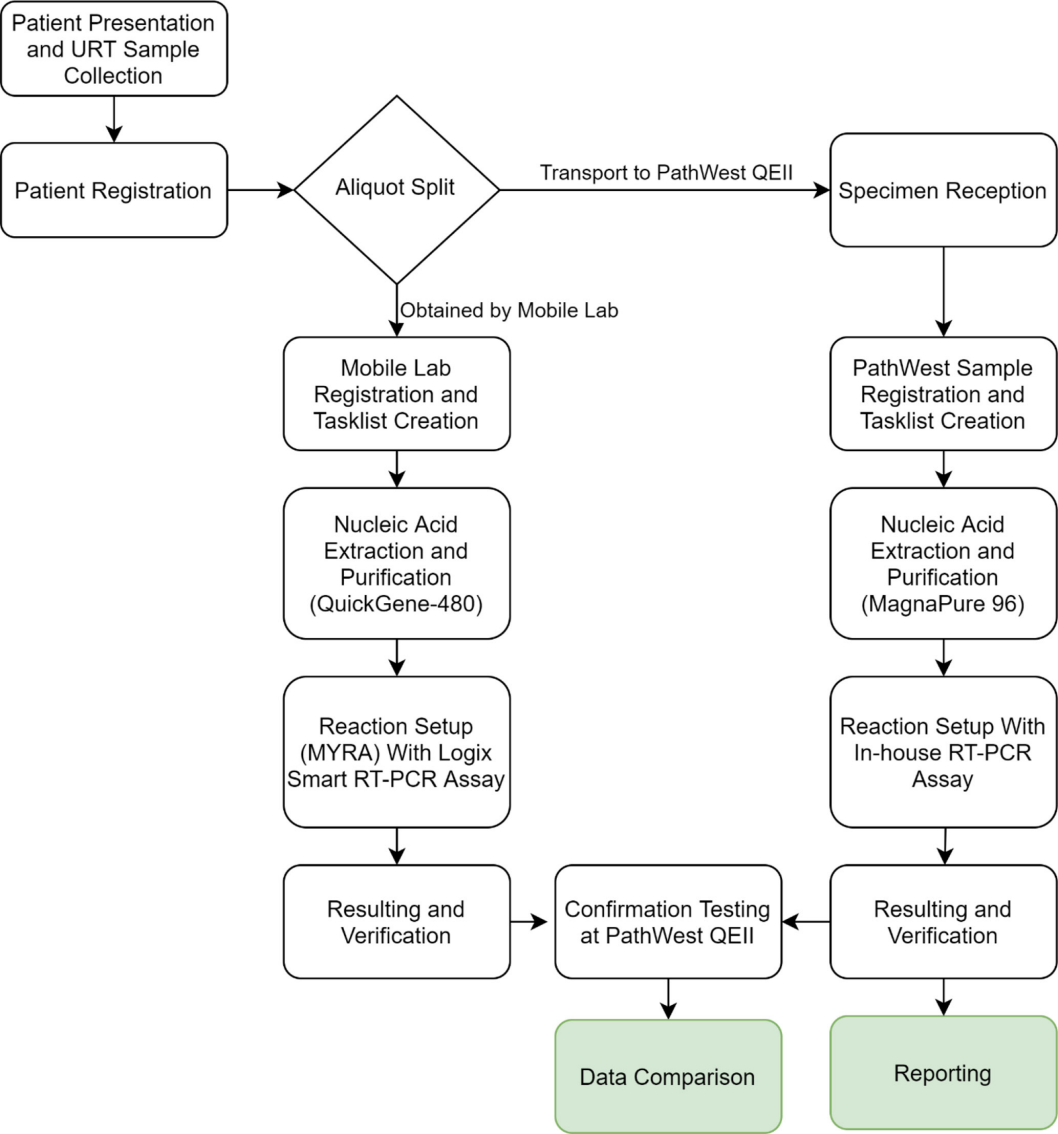
Workflow diagram for mobile laboratory operations in Derby and Broome. Confirmation testing occurred on return to Perth, comprising of repeating the obtained samples in the Smart Logix assay in parallel with additional inhibition testing.

The SARS-CoV-2 RT-PCR assay (Logix Smart COVID-19 Assay, Co-diagnostics) was used as per the manufacturer’s protocol, following extraction and purification steps performed using the QuickGene kit, using the modified protocol. Testing was performed on nasopharyngeal swabs obtained from any patient presenting to the designated collection area with upper or lower respiratory symptoms. The samples were split and dispatched for parallel testing by PathWest. For quality assurance purposes, all mobile laboratory samples were re-tested on return to Perth using fresh reagents. This was performed in parallel with MS2 RNA PCR in order to better assess sample inhibition. A flowchart illustrating on-deployment laboratory operations can be found in Fig. 1.

### Temperature control and logging

Reagents were transported in a conventional polystyrene cooler with flexible ice packs, pre-chilled at −20 °C for a minimum of 12 h prior to transit. Reagent temperatures for the Derby-Broome and Broome-Perth legs were recorded with a G^4^Medical data logger (Temprecord International, New Zealand). Temperature data were acquired every 2 min, and extracted from the instrument with Temprecord software (Temprecord International, New Zealand) on arrival at our destination. A temperature control failure threshold of 10 °C was set for this exercise, whereby the current in-use batch of reagents were to be discarded. To prevent bias in the data arising from reagent handling post-transit, the final 6 min of each record was removed from analysis.

## Results

### Quantification of SARS-CoV-2 RNA

After normalising the 10^−5^ quantification data, the mean absolute quantification values of a 10^−4^ equivalent dilution of the SARS-CoV-2 VCF extract was 309.25 copies µl^−1^ (σ=42.68) for the E gene target. No outliers were detected in this data set. An extrapolated undiluted sample copy number of 3 092 500 copies per microlitre formed the standard for sensitivity and limit of detection calculations (see Table 1).

**Table 1.** ddPCR quantification results for E gene target using the IHD-RT-PCR assay

IHD-RT-PCR (E gene)	10^−4^ (copies µl^−1^)	10^−5^ (copies µl^−1^)
Replicate 1	294	39.9
Replicate 2	263	32.1
Replicate 3	301	34.6
Replicate 4	286	26.4
Mean	286	33.3
Standard Deviation	16.5	4.9

ddPCR, droplet digital PCR; IHD-RT-PCR, in-house developed reverse transcriptase PCR.

### Extraction method comparison

For the QuickExtract kit, inhibition was evident when compared to QuickGene-extracted samples. SARS-CoV-2 positive, undiluted RNA extracted by this method did not amplify in the Logix Smart master mix in any replicate. A minimum 10-fold dilution in molecular grade water was required in order to overcome inhibition and restore consistent Cq intervals. For the QuickGene RNA Tissue II Kit, the consistent intervals of Cq values obtained from the dilution series suggested minimal inhibition was present. Cq values were also consistent between replicates throughout the entire dilution range.

### Lower limit of detection

**Fig. 2.**
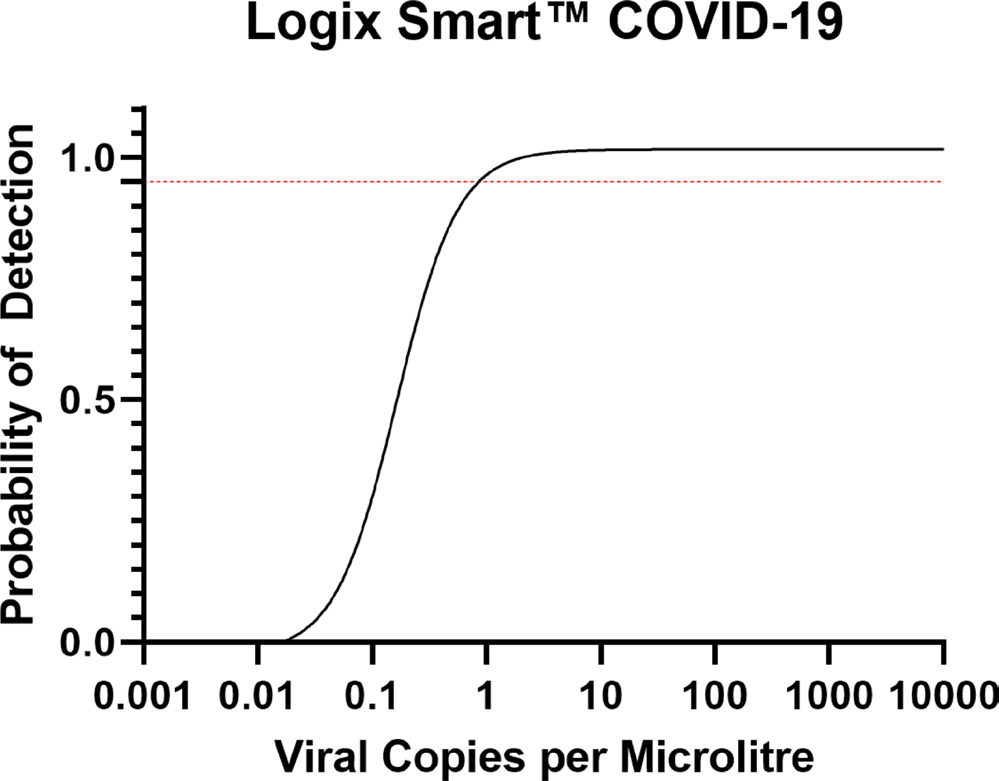
Probit regression for LLOD determination. Red dashed line indicates 95% probability of detection.

**Fig. 3.**
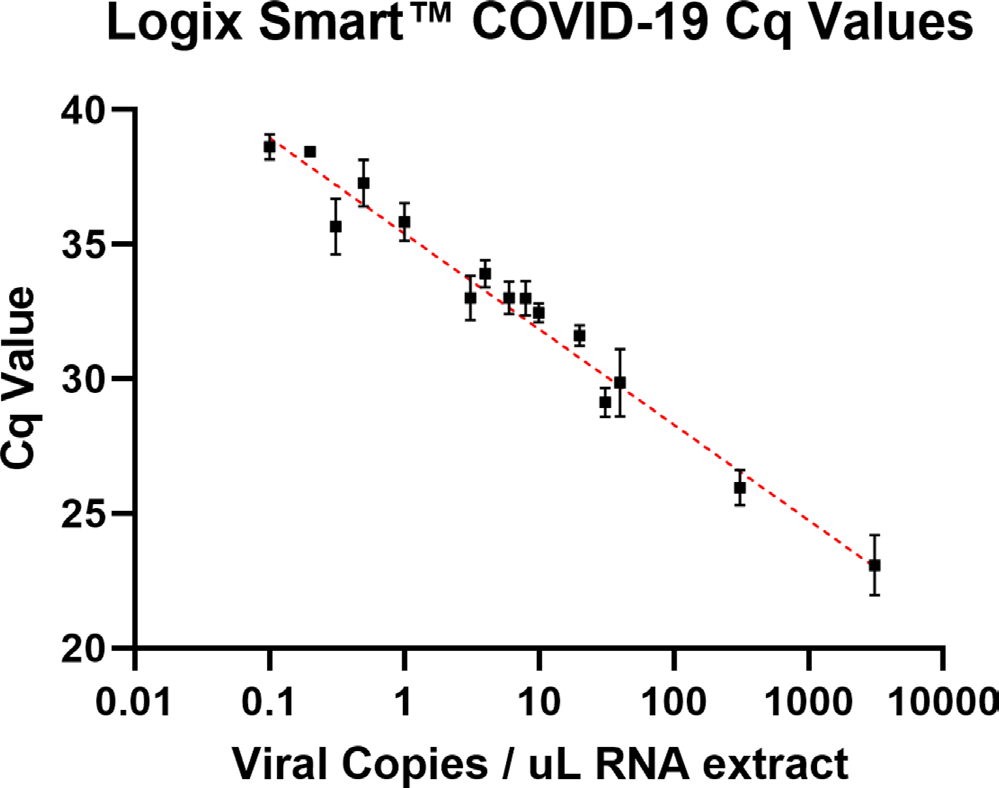
Semi-logarithmic regression of Cq values obtained from the Logix Smart COVID-19 assay using a titrated, quantified SARS-CoV-2 standard (R^2^=0.9289, error bars: standard deviation).

For the Logix Smart COVID-19 assay, the 95 and 99% LLOD for the RdRp target, in copies per microlitre of RNA extract were; 1.01 (upper 95 % CI: 2.04) and 2.80 (upper 95 % CI: 9.219) respectively, lower than the manufacturer’s sensitivity claim of 9.35 copies µl^−1^ [10] (see Fig. 2). These data were generated from a linear dilution series of the standard at the limit of detection (40 copies µl^−1^ to 0.003 copies µl^−1^). Across all data, there was strong inverse correlation (semi-log fit R^2^=0.9289) between calculated viral copies per microlitre and obtained Cq values (see Fig. 3).

### Cross reactivity

Low efficiency (approximately 0.55) cross reactivity with SARS-CoV-1 RNA was identified in the Logix Smart COVID-19 assay. No definitive cross reactivity was detected in the other 55 samples tested, including non-sarbecovirus coronaviruses OC43, HKU1 and NL63.

### Diagnostic test performance calculations

Two samples returned a false negative result at 1 in 5 dilution, however the errors resolved with repeat testing from undiluted specimen in parallel with the IHD-RT-PCR as per the error discrepancy algorithm. For included samples (*n*=207), there was perfect concordance with the IHD-RT-PCR assay; test accuracy was 100% (95% CI 98.23–100); sensitivity 100% (95% CI, 96.48–100); specificity 100 % (95% CI, 96.52–100), positive predictive value 100%, negative predictive value 100%.

### Reagent temperature tracking

**Fig. 4.**
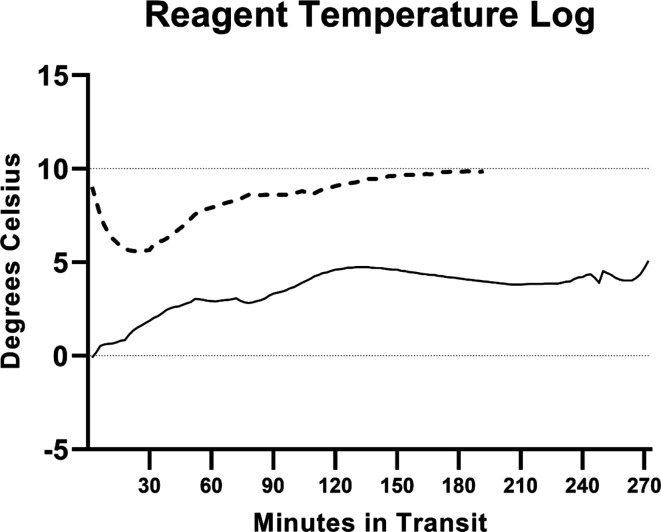
Temperature logs for the ex-Derby (dashed line) and ex-Broome (solid line) transit legs of the deployment.

Container temperatures did not exceed the critical threshold of 10 °C for either the Derby-Broome leg by road (x̃=8.49 °C, σ=1.53 °C) or the Broome-Perth leg by air (x̃=3.61 °C, σ=1.20 °C) (see Fig. 4). Container temperatures approached the critical threshold for the Derby-Broome leg, and visual inspection of the reagents on arrival showed that the reaction mix and control material had partially thawed. For the Broome-Perth leg, container temperatures did not exceed 5 °C, with no thawing observed in any reagent on arrival.

### Derby results

In total 16 patient samples were analysed over four runs. All samples tested were flocked swabs in universal transport medium (UTM) (Copan Diagnostics, USA). All quality controls passed; no contamination was present in the negative template controls (NTC) or extracted negatives; the Logix Smart internal control indicated no significant inhibition in any sample; and the positive controls were within range, Cq x̅=32.1, σ=1.04. SARS-CoV-2 was not detected in any patient samples, consistent with results obtained from PathWest.

### Broome results

For this 24 patient samples were analysed over two runs of which 20 were dry swabs, and four were flocked swabs in UTM. All quality controls passed: no contamination was present in NTC or extracted negatives; the Logix Smart internal control indicated no inhibition in any sample; and positive controls were within range, Cq x̅=28.31, σ=0.38. SARS-CoV-2 was not detected in any patient samples, consistent with results obtained from PathWest.

### Re-test results

On returning to Perth, all samples were re-tested at PathWest in Perth using the same method in parallel with an MS2 inhibitor control assay. All SARS-CoV-2 negative results were reproduced in the Logix Smart assay. All samples were positive for RnaseP, indicating the extracts were not inhibitory in this assay (Cq x̅=27.84, σ=2.75). Partial inhibition of the MS2 assay was evident in one sample (MS2 Cq: 37.64), although this was not evident in the Logix Smart internal control (RnaseP Cq: 22.15). Aside from this outlier, MS2 Cq values were consistent (Cq x̅=30.51, σ=0.56), with results from patient samples comparable to those obtained from negative controls (Cq x̅=30.52, σ=0.34) indicating that levels of inhibitors were low overall.

### Variation in positive control cq

All MIC runs containing Logix Smart assay data (i.e. sensitivity, specificity, patient validation and field trial data) were imported into a single project file in order to calculate the intra-assay coefficient of variation (CV) for the positive controls. A total of 37 replicates were performed from four separate aliquots of control material. The mean Cq value obtained was 28.18 (σ=1.87; CV=6.64 %). When the data were isolated, consistency was worse for positive controls used during the field deployment phase (Cq x̅=30.21, σ=2.12; CV=7.03 %).

## Discussion

There is an evident need for a reliable SARS-CoV-2 assay to extend the reach of centralised molecular diagnostic laboratories and bridge current diagnostic and logistic gaps. We refined the initial selection of equipment and reagents to design a standardised and reproducible procedure. This resulted in a reliable, safe and portable molecular diagnostics workflow. The final selection comprised a centrifuge-free pressure manifold for extraction and purification of coronavirus RNA, the Logix Smart COVID-19 RT-PCR assay for the coronavirus RDRP gene, the Myra compact fluid handling robot and the MIC thermal cycler.

The MYRA liquid handler performed well in this study. The platform was demonstrably robust, rapidly deployable, and did not require extended re-calibration after transit. Similarly, we found the Logix Smart RT-PCR assay to be well suited to the requirements of the mobile laboratory. Despite its functional simplicity, it exhibited similar analytical sensitivity to both the IHD-RT-PCR assay, and the GeneXpert SARS-CoV-2 assay currently deployed throughout regional Western Australia (0.56 copies µl^−1^) [15]. While cross reactivity with SARS-CoV-1 was evident, due to this virus being extinct in the wild, the impact of such a theoretical misdiagnosis is clinically unimportant.

Our mobile laboratory differs in scope to many near point-of-care systems. *In situ* testing in many peripheral laboratories generally relies on closed-format cartridge-based systems i.e. GeneXpert (Cepheid, Sunnyvale, CA), Luminex ARIES (Luminex Corp., Austin, TX, USA), ID Now (Abbott, Chicago, IL, USA), Simplexa (DiaSorin, Saluggia, Italy), or FilmArray (BioFire, Salt Lake City, UT, USA). While many of these options are better suited for operation by non-specialist clinical laboratory staff, they are generally unable to effectively respond to surge testing requirements or changes in assay targets. Almost all require investment in specialist hardware that works only for a single cartridge supplier. Further, with increasing global demand for laboratory plant and reagents, reliance on such closed commercial systems puts rapid public health responses in a precarious position if supply cannot match testing needs.

By opting for an open molecular diagnostics platform, we delivered flexible throughput, and the option to use alternative assays should another target be required. The open-platform MIC and Myra systems fulfilled these requirements. Furthermore, as they are locally built, they gave us improved supply chain security for consumables compared to many international suppliers. Supply chain disruption is a pervasive threat in this pandemic. We had originally planned to evaluate a second SARS-CoV-2 RT-PCR assay (Liferiver, Shanghai ZJ Bio-Tech Co., China), but due to supply disruption we were unable to procure reagents beyond an initial shipment of 100 reactions. Open platforms are not dependent on dedicated proprietary test kits, and thus facilitate the ability to validate and subsequently use any compatible assay. This allows the operators to employ a back-up test should the supply chain for primary test reagents fail. Additionally, the open nature of the MIC and Myra guarantees utility well beyond the life of the current pandemic. The systems are easily adapted to perform routine molecular diagnostics in regional laboratories, potentially reducing the reliance on centralised molecular testing into the future.

We aimed to avoid conventional centrifugation- or vacuum-based nucleic acid extraction kits for use in branch laboratories or austere locations, as these are dependent on heavy, bulky external devices for operation e.g. centrifuges and vacuum pumps. These devices reduce portability, can slow deployment, and draw a large amount of electricity which may not be appropriate where power is provided by solar panels, batteries or generator. The QuickGene Mini-480 pressure manifold used for sample processing weighed approximately 3 kilograms, has a small footprint suitable for use inside a small biological safety cabinet, a low peak power draw of six watts, and a throughput of 48 extractions per 45 min, fulfilling the requirements for the mobile laboratory.

Field trials of the mobile laboratory demonstrated consistently satisfactory test performance in two regional laboratory locations during a week-long regional deployment. The RT-PCR workflow we developed was operational within an hour of arrival in the regional laboratories, establishing an immediate increase in COVID-19 test capacity from four patient samples to 32 simultaneous samples per test run, and an estimated capacity of 150 samples per day. Allowing for controls, this workflow is capable of running a COVID-19 RT-PCR screening assay on batches of up to 36 patient samples at a time, producing a valid result 2 h after specimen receipt. Using staggered specimen preparation, it will be possible to process up to 150 screening assays per platform per day with two operators. Further deployment forward to a pathology specimen collection centre is a logical next step but has yet to be assessed.

### Limitations

The Logix Smart COVID-19 assay uses human RnaseP as an internal inhibitor control target. As with many current EUA FDA approved assays use of patient RNA for inhibition control is subject to variation due to differences in the number of human cells harvested. This is directly reflective of collection sites (e.g. nasopharyngeal vs. nasal) and / or quality of collection technique (depth of sample, *in situ* rotation of swab). To overcome this limitation, QuickGene LRT buffer was spiked with a standardised titre of MS2 coliphage. This allowed the user to confirm cases of genuine inhibition with a second PCR reaction. By running the MS2 assay in parallel with the Logix Smart assay, we are able to distinguish genuine inhibition from poor sample collection. Incorporating this secondary inhibitor control may be beneficial in preventing the reporting of false negative results in convalescent patients in situations where primary sample collection is sub-optimal.

There was some variability in assay performance on deployment. The calculated coefficient of variation (7.03%) exceeded the 5 % limit considered acceptable by in-house guidelines. Data obtained in Derby suggest that positive control Cq values increased with freeze-thaw cycles, which resolved on use of fresh reagents. Partial thawing of the control material during transit is likely to have further contributed to reagent degradation. This suggests that, when transit times exceed 1–2 h, cold chain methods require strengthening, particularly in hot climates. Although the temperature control failure threshold was not crossed during this exercise, the degradation observed suggests that the limit should be revised. Furthermore, our transit times were relatively short, not exceeding 4 h. Deployment to many remote regions of regional Australia is only possible by road, extending travel times at temperatures that often exceed 30 °C year-round, making maintenance of sub-zero temperatures problematic. Ideally, road transport of reagents should occur on dry ice in a double insulated container, however, the scarcity of dry ice in remote regions is a major hindrance to this approach. In order to rectify this limitation, we are currently investigating sub-zero eutectic phase change materials in combination with low-power portable refrigeration to assist in maintaining suitable temperatures for extended periods. This will ensure more robust on-deployment performance should extended transit times be necessary.

Automatic normalisation and thresholding also contributed to variation in reported inter-run Cq results for positive controls. For field testing, in the absence of internally calculated measurement uncertainty (MU) data, we relied on the Cq cut-off values supplied by Co-Diagnostics for quality assurance purposes. In order to align with Australian National Pathology Accreditation Advisory Council Tier 3B standards [16], we have implemented and are currently evaluating a method to calculate ongoing MU for positive and internal controls. MIC software automatically normalises PCR data for analysis based on intra-run peak fluorescence. Due to this, we found fixed thresholding and direct inter-run comparisons of control Cq inappropriate for this task. However, by performing retrospective analyses of control material from previous runs, concatenated and normalised together, ongoing MU can be calculated based on periodic quality control results. Automation of this protocol, for instance in a VBA enabled Microsoft Excel Worksheet updated daily with QC data, could form the foundation for a quality assurance framework for ongoing testing.

## Conclusion

The final version of the SARS-CoV-2 RT-PCR assay workflow was validated under the Australian Public Health Laboratory Network’s supervision in accordance with the terms and conditions of the Therapeutic Goods Administration’s emergency approval, to meet PathWest standards and eventual National Association of Testing Authorities quality standards. This workflow has comparable sensitivity and specificity to the PathWest assay. The mobile laboratory is designed to operate in a regional clinical laboratory with a class II biosafety cabinet, using equipment that can be transported throughout the state. We envisage operation of this RT-PCR assay using an equipment fleet staffed and supplied to meet varying regional test demands under the guidance of the public health emergency operations centre for the duration of the pandemic. At this point, we make no claims for operation in austere locations, though use of a portable safety cabinet and a specimen label generator would allow operation in State Health collection centres co-located with remote clinics.
